# Supplementary material for: Effects of the Inclusion of Dietary Bitter Gourd (Momordica charantia) on the Performance and Carcass Characteristics of Pigs: Potential Application in the Feed Chain
Source: Animals (Basel). 2023 Jun 30;13(13):2159. doi: 10.3390/ani13132159 (PMC10339919; doi:10.3390/ani13132159)
Supplement: Supplementary file 1 [file animals-13-02159-s001.zip › animals-2376760-supplementary.pdf]

Supplementary Table S1. Composition (%) and calculated nutrient levels (g/kg) of the experimental basal diets

**Ingredients (%)**	**Diet 25 - 50 kg**	**Diet 50 - 80 kg**	**Diet 80 - 120 kg**
Barley	33.50	19.90	15.05
Wheat	21.76	41.39	44.78
Wheat middlings	5.98	11.94	17.72
Corn	12.95	5.00	5.00
Soybean meal	7.38	1.40	-
Rape seed meal 00	6.46	8.78	7.38
Oats	-	-	3.00
Molasses beet	1.00	1.00	1.67
Palm kernel expeller	-	3.58	1.99
Sunflower meal (33% CP)	-	-	1.00
Sunflower meal (37% CP)	4.52	3.51	-
Palm oil	0.30	0.30	0.30
Rape seed expeller	1.00	-	-
Monocalcium phosphate	0.50	-	-
Salt	0.36	0.34	0.34
Dummy	1.32	-	0.22
Lysine-HCl (79%)	0.49	-	-
L-Lysine (50% liquid)	-	0.66	0.57
Methionine (99%)	0.09	0.04	0.03
Threonine (98%)	0.16	0.13	0.12
Valine (99%)	0.01	-	-
Formic acid	0.14	-	-
Diamol	-	0.89	-
Lactic acid	0.49	-	-
Limestone	1.03	0.64	0.54
Lecithin	1.00	0.49	0.29
Phytase	0.004	0.004	0.003
**Calculated nutrients (g/kg)**			
Net energy (MJ/kg)	9.51	9.35	9.36
Dry matter	881.49	876.79	873.18
Ash	43.49	46.25	36.37
Crude protein	163.73	151.69	138.79
Fat	40.62	37.47	36.39
Crude fibre	45.51	51.90	51.30
Sugar	38.18	40.03	43.02
Starch	394.67	400.50	417.34
SID_LYSs	9.54	7.87	6.88
SID_METs	3.19	2.57	2.21
SID_M+Cs	5.72	5.07	4.61
SID_THRs	6.19	5.32	4.77
SID_TRPs	1.81	1.58	1.41
SID_ARGs	8.53	7.61	6.66
SID_ILEs	5.17	4.48	3.99
SID_VALs	6.43	5.68	5.16
SID_HISs	3.51	3.16	2.91
SID_ALAs	5.57	4.85	4.46
SID_ASPs	10.18	7.99	6.85
SID_GLUs	30.32	29.89	28.38
SID_GLYs	5.72	5.33	4.86
SID_LEUs	9.82	8.51	7.76
SID_PHEs	6.39	5.61	5.10
SID_PROs	10.68	10.31	9.85
SID_SERs	6.24	5.59	5.16
SID_TYRs	4.03	3.41	3.09

To reach the test levels of bitter gourd in the experimental diet B contained 99.375% of basal diet with 0.625% bitter gourd, diet C contained 98.75% of basal diet with 1.25% bitter gourd, diet E contained 99.32% of basal diet with 0.68% bitter gourd, diet F contained 98.64% of basal diet with 1.36% bitter gourd, diet H contained 99.35% of basal diet with 0.65% bitter gourd, and diet I contained 98.7% of basal diet with 1.3% bitter gourd.
